# Seroprevalence of *Toxoplasma gondii* in wild boars, red deer and roe deer in Poland

**DOI:** 10.1051/parasite/2015017

**Published:** 2015-05-20

**Authors:** Lucjan Witkowski, Michał Czopowicz, Dan Alexandru Nagy, Adrian Valentin Potarniche, Monica Adriana Aoanei, Nuriddin Imomov, Marcin Mickiewicz, Mirosław Welz, Olga Szaluś-Jordanow, Jarosław Kaba

**Affiliations:** 1 Laboratory of Veterinary Epidemiology and Economics, Faculty of Veterinary Medicine, Warsaw University of Life Sciences Warsaw Poland; 2 Faculty of Veterinary Medicine, University of Agricultural Sciences and Veterinary Medicine Cluj-Napoca Romania; 3 ERASMUS Student from Faculty of Veterinary Medicine, University of Agricultural Sciences and Veterinary Medicine Cluj-Napoca Romania; 4 Veterinary, Zootechnics and Lambling Faculty, Samarkand Agriculture University Uzbekistan; 5 Voivodeship Veterinary Inspectorate in Krosno Poland; 6 Division of Small Animal Infectious Diseases, Department of Small Animal Diseases with Clinic, Warsaw University of Life Sciences-SGGW Poland

**Keywords:** Toxoplasmosis, Wildlife, Game, Epidemiology, Meat juice, ELISA

## Abstract

Little is known about the prevalence of *Toxoplasma gondii* in wild life, particularly game animals in Poland. Meat juice collected during the 2009/2010 and 2010/2011 hunting seasons from 552 red deer (*Cervus elaphus*), 367 wild boars (*Sus scrofa*) and 92 roe deer (*Capreolus capreolus*) was tested for *T. gondii* antibodies using the multi-species ID Screen Toxoplasmosis Indirect kit (IDvet, Montpellier, France). Antibodies to *T. gondii* were detected in 24.1% of red deer (95% CI: 20.7%, 27.8%), 37.6% of wild boar (95% CI: 32.8%, 42.7%) and 30.4% of roe deer (95% CI: 22.0%, 40.5%). To the authors’ best knowledge, this is the first epidemiological report of *T. gondii* prevalence in red deer, roe deer and wild boars in Poland. *T. gondii* is present in wildlife animal tissues and consumption of the game may be a potential source of infection for humans.

## Abstract (fr)

On en sait peu sur la prévalence de *Toxoplasma gondii* chez les animaux sauvages, en particulier le gibier, en Pologne. Le jus de viande recueilli au cours des saisons de chasse 2009/2010 et 2010/2011 de 552 cerfs élaphes (*Cervus elaphus*), 367 sangliers (*Sus scrofa*) et 92 chevreuils (*Capreolus capreolus*) a été testé pour les anticorps de *T. gondii* en utilisant le kit indirect de toxoplasmose multi-espèces ID Screen (IDvet, Montpellier, France). Des anticorps dirigés contre *T. gondii* ont été détectés chez 24.1 % des cerfs élaphes (IC à 95 % : 20.7 %, 27.8 %), 37.6 % des sangliers (IC à 95 % : 32.8 %, 42.7 %) et 30.4 % des chevreuils (IC à 95 % : 22.0 %, 40.5 %). À la connaissance des auteurs, ceci est le premier rapport épidémiologique de la prévalence de *T. gondii* chez le cerf, le chevreuil et le sanglier en Pologne. *T. gondii* est présent dans les tissus des animaux sauvages et la consommation de gibier peut être une source potentielle d’infection pour l’homme.

## Introduction

The protozoan *Toxoplasma gondii* infects a wide range of mammal and avian species. Infection in humans may occur through the ingestion of uncooked or undercooked meat containing tissue cysts, through the ingestion of food or water contaminated by oocysts excreted in feline feces, and by mother-to-child transmission during pregnancy. *T. gondii* infection is common in many domesticated and wild animals used for food production and the European Food Safety Authority (EFSA) has recommended the surveillance and monitoring of toxoplasmosis in humans, animals and foodstuffs. There are numerous surveys worldwide documenting the prevalence of *T. gondii* in food animals. Compared with domestic livestock species, little is known about *T. gondii* prevalence in wildlife, particularly in Poland [10, 12, 13, 21, 31].

The aim of the study was to assess the seroprevalence of *T. gondii* in carcasses of wild boar, red deer and roe deer intended for human consumption in Poland.

## Materials and methods

The study was approved by the 3rd Local Commission for Ethics in Animal Experiments (Decision No. 44/2009). Wildlife animal population size was estimated on the basis of the results of monitoring carried out by the Polish Hunting Association in the 2009/2010 hunting season. Estimated population sizes of wild boars, roe deer and red deer were 250 000, 757 000 and 145 000 individuals, respectively. During this season, 197 000 wild boars, 162 000 roe deer, and 41 100 red deer were hunted [6]. A minimum sample size of 97 was determined for each animal species in order to estimate the prevalence with at least 10% precision at 50% expected prevalence and 95% level of confidence. The calculations were performed in EpiTools [30]. The 95% confidence intervals (95% CI) were calculated for prevalence using the Wilson score method [1]. According to Polish legal regulations, all carcasses of hunted animals are collected by several authorized companies. The animals were hunted in various regions, in 12 of 16 voivodships of Poland. The samples of meat from masseter tissue were obtained from carcasses accepted for human consumption collected in facilities belonging to two companies during hunting seasons 2009/2010 and 2010/2011. All samples were stored at −20 °C until testing. Thawed samples (approx. 1 g) were centrifuged and the meat juice was tested using a commercially available ELISA test (the multi-species ID Screen^®^ Toxoplasmosis Indirect kit, IDvet, Montpellier, France) according to the manufacturer’s instructions.

## Results and discussion

*T. gondii* antibodies were detected in 24.1% (133/552) of red deer (95% CI: 20.7%, 27.8%), 37.6% (138/367) of wild boar (95% CI: 32.8%, 42.7%) and 30.4% (28/92) of roe deer (95% CI: 22.0%, 40.5%).

This is the first epidemiological report of *T. gondii* prevalence in red deer, roe deer and wild boars in Poland. These results show that *T. gondii* is widespread in game from Poland. The seroprevalence of *T. gondii* in wild boar in Poland (37.6%) is similar to recent data from Latvia (33%) [11] and Finland (33%) [19]. It is much higher compared to most European countries where it ranged from 6% to 25% [4, 5, 7, 9, 14, 23, 25, 26, 28]. Prevalence over 50% has been reported only twice in Europe [27, 34].

In addition, the prevalence of *T. gondii* in the Polish population of roe deer (30.4%) is relatively high compared to Italy (2.4%) [14] and Spain (14%) [24]. However, similar prevalence was described in Sweden (34%) [22] and even higher in France (46%) [8].

Little is known about prevalence of *T. gondii* in red deer in Europe. In the present study, 24.1% of investigated animals were positive. In other European studies, antibodies against *T. gondii* were found in 7.7% red deer in Norway [32], 15.6% in Spain [17] and 45% in the Czech Republic [3]; however no positive animals were found in Italy [14].

No attempt has been made to compare the results of this study with other worldwide reports because many factors could influence these results, e.g. variable densities of domestic cats and environmental oocyst contamination. Furthermore, age-related differences in seroprevalence have been reported in some studies [34]. There are also significant differences in numbers of investigated samples, e.g. a previous report from Poland described *T. gondii* in only three roe deer [31]. Moreover, publications differ in terms of samples tested and assays used. In the present study, like in many others [2, 5, 11, 27, 29], meat juice was used. Several methods have been proposed for the detection of antibodies to *T. gondii* and there is a wide range of serological assays available commercially. However, none is a gold standard [2, 16]. The modified agglutination test (MAT) has been most commonly employed but several ELISA kits have also been used previously to detect *T. gondii* antibodies. High agreement between MAT and ELISA has been documented in most investigated animal species and both tests are suitable for epidemiological studies [15, 18, 20, 33, 34].

## Conclusions

To the authors’ best knowledge, this is the first epidemiological report of *T. gondii* prevalence in red deer, roe deer and wild boars in Poland. It shows that *T. gondii* is present in wildlife animal tissues in Poland and their consumption may be a potential source of infection for humans.

## Conflict of interest

The authors declare that they have no conflict of interest.
